# Advancing high-performance visible light communication with long-wavelength InGaN-based micro-LEDs

**DOI:** 10.1038/s41598-024-57132-9

**Published:** 2024-03-25

**Authors:** Fu-He Hsiao, Wen-Chien Miao, Tzu-Yi Lee, Yi-Hua Pai, Yu-Ying Hung, Daisuke Iida, Chun-Liang Lin, Chi-Wai Chow, Gong-Ru Lin, Kazuhiro Ohkawa, Hao-Chung Kuo, Yu-Heng Hong

**Affiliations:** 1 Semiconductor Research Center, Hon Hai Research Institute, Taipei, 11492 Taiwan; 2 Department of Electrophysics, College of Science, National Yang Ming Chiao Tung University, Hsinchu, 30010 Taiwan; 3 Department of Photonics and Institute of Electro-Optical Engineering, College of Electrical and Computer Engineering, National Yang Ming Chiao Tung University, Hsinchu, 30010 Taiwan; 4 Computer, Electrical and Mathematical Sciences and Engineering (CEMSE) Division, King Abdullah University of Science and Technology (KAUST), 23955 6900 Thuwal, Saudi Arabia; 5 Department of Electrical Engineering, Graduate Institute of Photonics and Optoelectronics, National Taiwan University, Taipei, 10617 Taiwan

**Keywords:** Inorganic LEDs, Fibre optics and optical communications, Optoelectronic devices and components

## Abstract

This study showcases a method for achieving high-performance yellow and red micro-LEDs through precise control of indium content within quantum wells. By employing a hybrid quantum well structure with our six core technologies, we can accomplish outstanding external quantum efficiency (EQE) and robust stripe bandwidth. The resulting 30 μm × 8 micro-LED arrays exhibit maximum EQE values of 11.56% and 5.47% for yellow and red variants, respectively. Notably, the yellow micro-LED arrays achieve data rates exceeding 1 Gbit/s for non-return-to-zero on–off keying (NRZ-OOK) format and 1.5 Gbit/s for orthogonal frequency-division multiplexing (OFDM) format. These findings underscore the significant potential of long-wavelength InGaN-based micro-LEDs, positioning them as highly promising candidates for both full-color microdisplays and visible light communication applications.

## Introduction

Visible light communication (VLC) stands out as a promising and innovative wireless technology that harnesses visible light for high-speed data transmission, positioning itself as a potential successor to traditional wireless fidelity (Wi-Fi) and indoor wireless communication methods^1–4^. As the demand for faster internet data transmission continues to surge, the existing radio spectrum nears its capacity limits, prompting researchers to explore alternative avenues for data transmission. This exploration led to the inception of VLC, which operates within the wavelength range of 380–750 nm. VLC, in essence, taps into previously untapped spectrum resources, offering a solution to the growing problem of frequency congestion. It holds the promise of delivering ample bandwidth, effectively addressing the bandwidth limitations that plague traditional radio frequency (RF) communications^5,6^. VLC comes with advantages such as data security, immunity to electromagnetic interference, no licensing requirements, and rapid response times^4^. A particularly noteworthy feature of VLC is its ability to provide illumination while concurrently transmitting data. This dual functionality not only reduces operational costs but also curtails unnecessary power consumption, making it an energy-efficient choice. The potential applications of VLC are vast and encompass a wide array of fields, ranging from automotive and indoor networks to mobile location services^4,7,8^. Its compatibility with environments that prohibit radio waves further enhances its versatility and applicability. Presently, VLC is making its mark in various facets of everyday life, and as its popularity continues to grow, it holds the potential to become a dominant player in numerous communication applications. Among these, micro light-emitting diodes (micro-LEDs) emerge as exceptional light sources for VLC^9–11^. InGaN-based micro-LEDs, in particular, emerge as strong contenders for next-generation full-color VLC technology, thanks to their remarkable attributes such as high brightness, rapid response times, low power consumption, and exceptional color modulation capabilities. These qualities position InGaN-based micro-LEDs at the forefront of the evolution of VLC technology, promising to revolutionize the landscape of high-speed, data-centric communication^12,13^.

In recent years, InGaN-based micro-LEDs have garnered significant attention due to their versatile applications; however, despite their capacity to achieve full-color illumination by adjusting indium content, there is a downside to consider. In long-wavelength InGaN-based micro-LEDs featuring a high indium content, the pronounced quantum confined Stark effect (QCSE) adversely affects their performance^14–16^. This is primarily due to the strain-induced polarization stemming from lattice mismatch, resulting in a reduced overlap of the electron–hole wave functions, which consequently diminishes not only the radiative recombination rate and luminous efficiency but also leads to a reduction in bandwidth^15,17,18^. A sufficient modulation bandwidth is crucial for achieving high-speed VLC. Furthermore, a minimal wavelength shift and a narrow full-width at half maximum (FWHM) play a vital role in preventing crosstalk between different channels when implementing wavelength division multiplexing^19^. Recent efforts have focused on developing highly efficient and high-transmission-performance InGaN-based micro-LEDs within the yellow-green to red emission wavelength range. Zhu et al.^20^ showcased a series connection of micro-LED arrays that boosted output power while maintaining optical bandwidth, resulting in green- and yellow-emitting micro-LED devices achieving maximum − 3 dB bandwidths of 348.1 MHz and 93.9 MHz, respectively. Lin et al.^21^ introduced a semipolar green micro-LED designed to suppress the QCSE effect and enhance emission output power, achieving an impressive − 3 dB bandwidth of 800 MHz with data rates exceeding 1.5 Gbit/s. Additionally, Huang et al.^22^ demonstrated a high − 3 dB bandwidth yellow-green InGaN-based micro-LED by utilizing nanoporous distributed Bragg reflectors (DBR) to enhance light extraction efficiency and serve as a strain-released layer, effectively mitigating the QCSE effect. Furthermore, Huang et al.^23^ presented red-emitting InGaN-based micro-LEDs with high efficiency and exceptional modulation bandwidth, achieved through the adoption of atomic layer deposition (ALD) passivation techniques, the incorporation of a superlattice structure, and the integration of DBR technology. In our previous work^24^, we demonstrated the potential of InGaN-based red micro-LEDs featuring a single quantum well (SQW) for applications in VLC. Reducing the number of quantum well (QW) layers not only improved the crystal quality but also enhanced data transmission rates.

In this study, we fabricated long-wavelength micro-LED arrays for VLC applications. Both yellow and red micro-LEDs are based on the same epitaxial hybrid QWs structure, simply by adjusting the indium content within the InGaN QWs. We then conducted a comparative analysis of the structural and optoelectronic characteristics to highlight the distinctions between the yellow and red micro-LEDs. Notably, the 30 μm × 8 micro-LED arrays exhibited outstanding external quantum efficiency (EQE) and exceptional transmission performance. The InGaN-based yellow micro-LEDs achieved a maximum − 3 dB bandwidth of 630 MHz, while the red ones reached 418 MHz. Furthermore, the yellow micro-LED arrays achieved a data rate of 1.5 Gbit/s when employing the orthogonal frequency-division multiplexing (OFDM) format. These findings highlight the significant potential of long-wavelength InGaN-based micro-LEDs, poised to advance high-speed VLC and microdisplay technologies, promising transformative opportunities in diverse technological domains.

## Materials and methods

**Figure 1:**
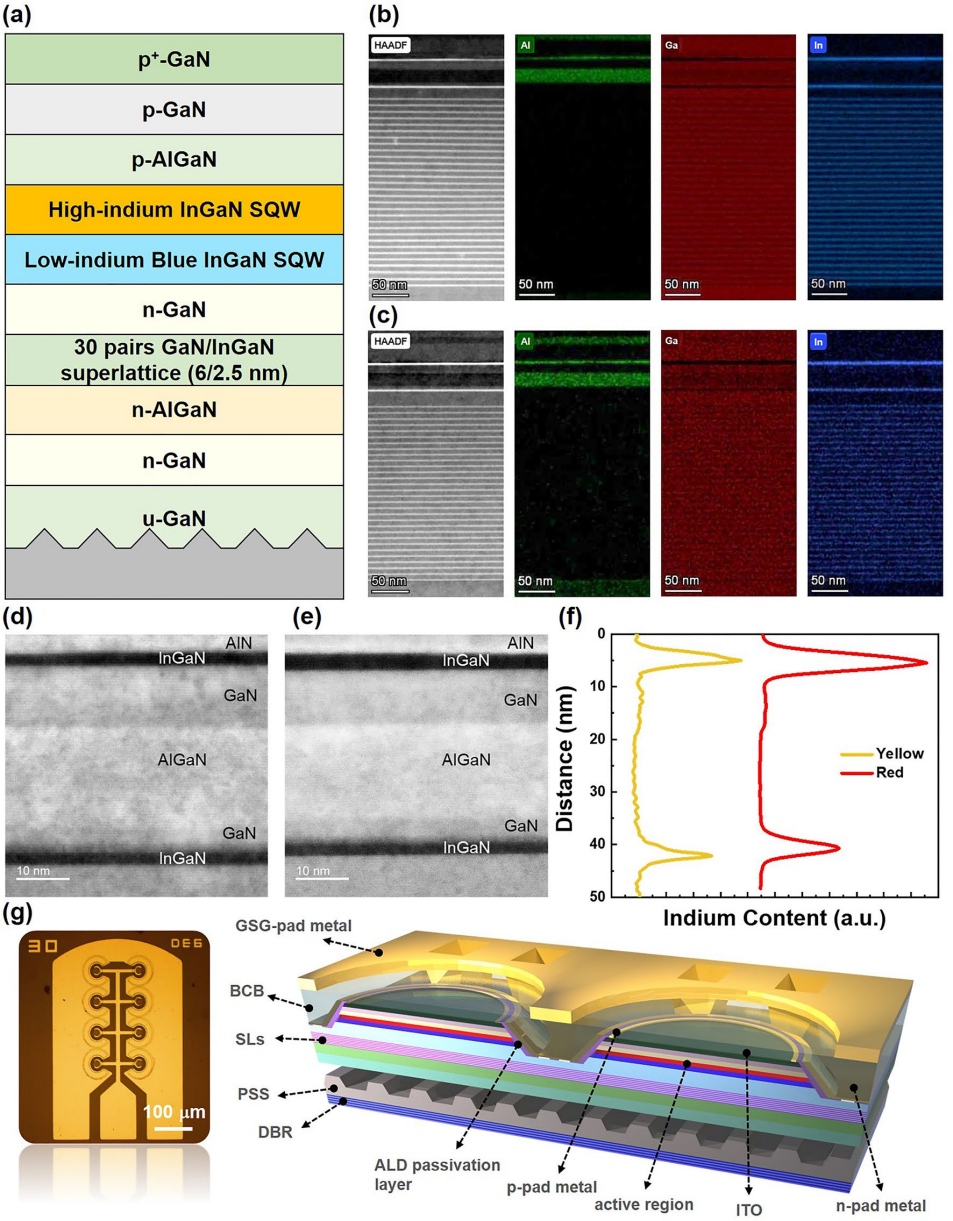
Material analyses and the proposed micro-LED device. (**a**) Schematic epitaxial structure of the micro-LED samples. (**b**,**c**) HAADF-STEM images and EDS mappings of the Al, Ga, and In elements in the yellow and red micro-LED devices, respectively. (**d**,**e**) TEM images of hybrid QWs structure for yellow and red samples, respectively. (**f**) The EDS line scan analysis. (**g**) Optical microscope image and corresponding schematic diagram of the proposed micro-LED device.

Figure 1a displays a schematic of InGaN-based long-wavelength-emission epitaxial with hybrid QWs structure. Both red and yellow samples were grown on 2-inch *c*-plane patterned sapphire substrates (PSS) using metal organic vapor phase epitaxy (MOVPE) technology. First, a 2-μm-thick unintentionally doped (uid) GaN layer was grown on PSS. Next, Si-doped n-GaN with a thickness of 8 μm and Si-doped n-AlGaN with a thickness of 1.7 μm were deposited, where n-AlGaN was used as the contact layer for n-type metals due to low electrical resistance^25^. Afterward, 30 periods of GaN (6 nm)/ In_0.08_Ga_0.92_N (2.5 nm) superlattices (SLs) structure were inserted to alleviate the effect of QCSE and improve the crystal quality of the following InGaN QW, followed by a n-GaN layer. Subsequently, a hybrid QWs structure consisting of a low-indium blue InGaN SQW and a high-indium InGaN SQW were grown. Low-indium-content InGaN, uid-GaN, and n-AlGaN hole-blocking barrier layers are included in the low-indium blue InGaN SQW. Meanwhile, the high-indium InGaN QW is composed of high-indium-content InGaN as the active layer and AlN/GaN barrier layer. Finally, p-AlGaN, Mg-doped p-GaN, and a heavily Mg-doped p^+^-GaN contact layer were grown. Figure 1b,c present the cross-sectional high-angle annular dark-field (HAADF)-scanning transmittance electron microscopy (STEM) and the energy-dispersive x-ray spectroscopy (EDS) mapping images for the yellow and red samples, respectively. The results confirm that there are 30 pairs of SLs structure, blue SQW with relatively low indium content, and SQW with relatively high indium content in both epitaxial samples. Furthermore, we concentrate on the hybrid QWs structure, as illustrated in Fig. 1d,e, where the TEM images reveal a similar structure between the two samples. In addition, the EDS line scan analysis in Fig. 1f confirms that the high-indium InGaN QW in the red sample indeed contains higher indium content than in the yellow one.

In this paper, we fabricated 30 μm × 8 micro-LED arrays, employing a suite of six core technologies depicted in Fig. 1g to optimize their performance for VLC. The first technique focuses on minimizing the emitting size by designing mesas with a 30 μm diameter. This choice aimed to reduce the impact of the RC time constant on bandwidth, primarily constrained by the spontaneous radiative recombination lifetime of carriers and the RC time constant. Second, we designed a circular mesa platform with C-shaped toroidal electrodes to enhance current spreading, enabling higher current density operation. In the third technique, we designed a parallel array of micro-LEDs to increase the optical output power and improve the signal-to-noise ratio (SNR). According to the previous study, the parallel design of micro-LEDs has little effect on the RC time constant, so that the same level of bandwidth can be maintained while increasing the optical power. Next, in the process, we use ALD passivation layer technology to help reduce the sidewall defects caused by dry etching of small-size LEDs. Defects caused by trap-assisted tunneling currents and non-radiative recombination reduce the concentration of effective carriers in the QW, which increases the radiatively recombined carrier lifetime and improves efficiency while improving the SNR in the communication performance. In addition, we adopt the Benzocyclobutene (BCB) planarization to enlarge the electrode area more effectively in the same die area, avoiding the wire melting due to the rapid temperature rise caused by the current congestion under high current density. Last but not least, a distributed Bragg reflector (DBR) consisting of 5.5 pairs of SiO_2_/TiO_2_ layers was implemented to boost the light extraction efficiency of micro-LED devices.

## Results and discussion

### Structural analyses

**Figure 2:**
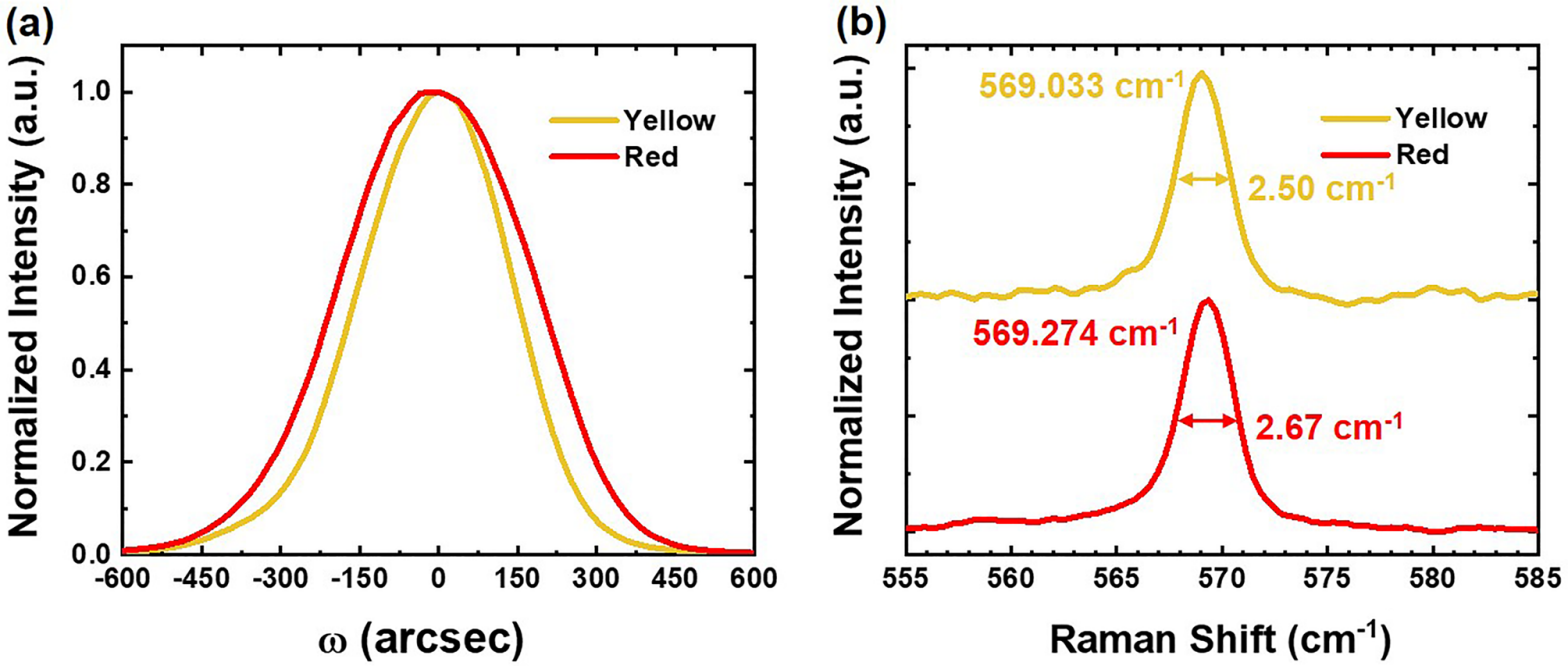
Spectra comparison between the fabricated yellow and red micro-LEDs. (**a**) The ω-scan rocking curves from (002) XRD measurement of yellow and red samples. (**b**) Measured Raman spectra of the E_2_ mode of yellow and red samples.

Generally, achieving longer wavelength-emission necessitates a higher indium content within the quantum well. This implies that a lower growth temperature is needed. However, the low-temperature growth process is associated with defect formation, and as the indium content rises, so does the density of these defects. To investigate the crystalline quality of yellow and red LEDs, we performed an X-ray diffraction (XRD) measurement on the epitaxial structure using the PANalytical X’Pert Pro (MRD) diffractometer. Figure 2a shows symmetric (002) ω-scan rocking curves of yellow and red LEDs. The FWHMs of the symmetric (002) rocking curves of yellow and red LEDs were 334.44 and 423.01 arcsec, respectively. In addition, we can access the dislocation density (*D*) through the use of the following expression^26,27^:

1 $$D=\frac{{\beta }^{2}}{2\pi {b}^{2}{\text{ln}}2}$$

where $\beta$ corresponds to the FWHM of the relevant diffraction peak, and *b* represents the absolute value of the Burgers vector. Upon calculation, we determined that the dislocation densities for the yellow and red LEDs were 2.244 × 10^8^ and 3.591 × 10^8^ cm^−2^, respectively. These findings serve to highlight a distinction between the two samples. Specifically, the red LEDs, which necessitate a higher indium content in the QW, exhibit a great number of dislocation defects and suffer from a more pronounced lattice mismatch.

We employed Raman spectroscopy as a valuable tool to assess the stress levels in InGaN-based micro-LEDs, specifically by measuring the high-energy Raman E_2_ mode. The experimental setup involved focusing a laser spot onto the surface of the epitaxial structure of p^+^-GaN, enabling the detection of accumulated strain within the QWs through Raman spectroscopy. The Raman signals originating from the samples are selectively filtered using the MS 5204i monochromator (SOL instruments) and subsequently captured by the DR-324B-FI-601 CCD detector (Oxford Instruments). The results depicted in Fig. 2b provide valuable insight into the material characteristics of our sample. We present the normalized Raman spectra of the E_2_ mode for both yellow and red LED samples. The position of the E_2_ mode peak is an exceptionally sensitive indicator of strain within a given layer, serving as a robust measure of the material strain state. In Fig. 2b, it is evident that both the yellow and red samples exhibit a noticeable redshift in the E_2_ peak when compared to stress-free GaN, which is typically observed at 567.36 cm^−1^^28^. This pronounced redshift strongly implies the presence of compressive strain in the InGaN-based micro-LEDs. Furthermore, the specific peak positions of the E_2_ mode for the yellow and red samples are documented as 569.033 cm^−1^ and 569.274 cm^−1^, respectively. These results suggest that the yellow sample experiences a relatively lower degree of compressive strain. Additionally, as shown in Fig. 2b, it is worth noting the difference in FWHM of the E_2_ Raman spectra between the two samples. The FWHM measured for the yellow sample is 2.50 cm^−1^, and for the red one, it is 2.67 cm^−1^. This disparity in FWHM values suggests that the yellow sample is likely better equipped to mitigate the accumulation of strain on its surface, potentially inhibit the generation of defects, and exhibit superior crystalline quality in the QW active layers.

### EL spectra and electrical properties

**Figure 3:**
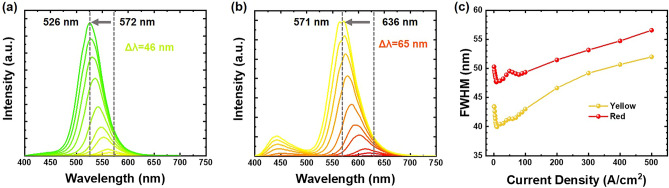
EL spectra at different current densities of (**a**) yellow and (**b**) red micro-LEDs. (**c**) FWHM-current density curves of micro-LEDs.

In this section, we conducted an in-depth exploration of the electroluminescence (EL) characteristics exhibited by the 30 μm × 8 micro-LED arrays. Figure 3a,b depict the EL emission spectra as we increment the injection current from 1 to 500 A/cm^2^. At the outset, with an initial injection current density of 1 A/cm^2^, the yellow emission wavelength was observed to commence at 572 nm. However, as the current density was incrementally raised to 500 A/cm^2^, a noteworthy shift in the emission wavelength became apparent, ultimately settling at 526 nm, accompanied by a corresponding FWHM of 52 nm. On the other hand, the red micro-LEDs undergo a more substantial wavelength shift, ranging from 636 to 571 nm, with a broader FWHM of 57 nm. The significant blue shift occurs as a result of the internal electric field in the *c*-plane InGaN QW being screened with increasing injection current density. Additionally, the considerable shift in wavelength can also be attributed to the band-filling of the localized state caused by fluctuations in indium content. These findings illustrate that the red micro-LEDs are more susceptible to QCSE due to their higher indium content. Regarding the linewidth, the nonuniform distribution of indium can generate spatial potential variations that trap carriers, leading them to transition between various energy levels, thereby broadening the spectra. The broader FWHM observed in red micro-LEDs indicates a greater presence of potential fluctuations, primarily due to the relatively nonuniform indium distribution. Consequently, the material quality of red micro-LEDs appears to be relatively inferior compared to the yellow ones, aligning with the observations from the XRD rocking curve and Raman spectra.

Besides, as depicted in Fig. 3b, we made an intriguing discovery related to the red micro-LEDs as the injection current was incrementally increased. There’s the emergence of additional emission peaks within the blue spectral range. Nonetheless, it is worth noting that the additional EL intensities were relatively weak compared with the primary EL emission. The appearance of the additional emission could arise from either the defects generated within the QWs or the overflow of holes into the blue SQW^29,30^. However, the yellow micro-LEDs, possessing an identical high-potential barrier structure and hole-blocking n-AlGaN barrier, do not exhibit these additional emission peaks. This dismisses the possibility of hole overflow being the primary cause of the phenomenon in the red micro-LEDs. Therefore, we propose that there exist defect areas within the QWs of red micro-LEDs characterized by lower indium content.

**Figure 4:**
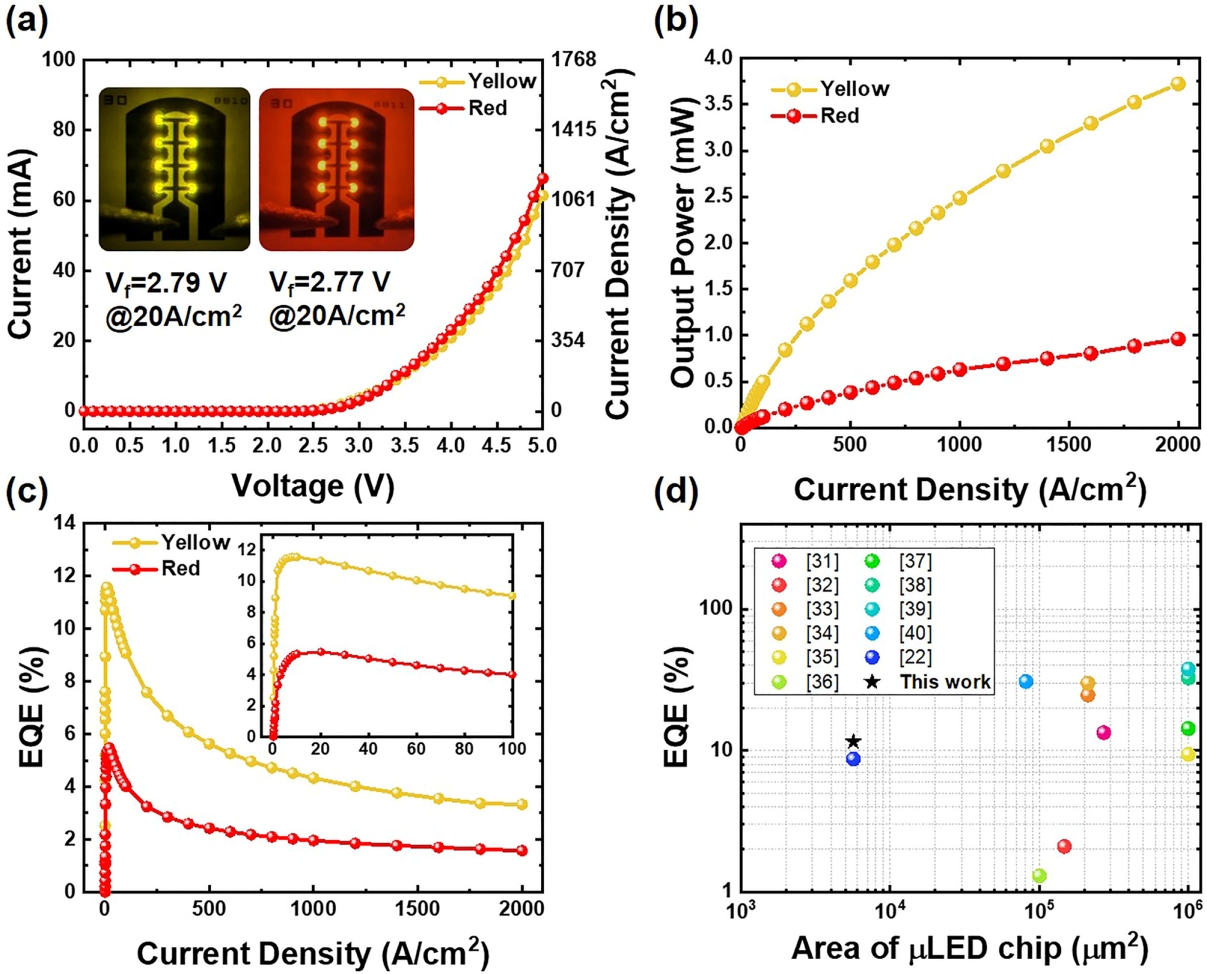
Micro-LEDs device performance. (**a**) Current–voltage characteristics. The insets show the illumination images. (**b**) Optical output power as a function of current density. (**c**) EQE-current density curves of micro-LEDs. (**d**) benchmark of EQE values for InGaN-based yellow micro-LEDs.

The electrical properties of micro-LEDs are summarized in Fig. 4. The current–voltage (*I*–*V*) curves are shown in Fig. 4a, with the accompanying insets showing the illumination images of both micro-LEDs at an injection current density of 20 A/cm^2^. At this current density, the forward voltage for yellow and red micro-LEDs are 2.79 V and 2.77 V at 20 A/cm^2^, respectively. This aligns with our expectations, as red micro-LEDs typically possess a narrower energy bandgap, resulting in a correspondingly lower forward voltage. As depicted in Fig. 4b, there is a linear relationship between current density and optical output power until the output power reaches 0.5 mW. Beyond this point, the self-generated thermal effect begins to exert a notable impact on the device, leading to a gradual saturation in optical power. Figure 4c demonstrates the EQE values in relation to injection current density, where the maximum EQE values achieved are 11.56% and 5.47% for yellow and red micro-LEDs, respectively. Figure 4d summarizes the EQE values plotted against the emission area for yellow micro-LEDs within the wavelength range of 550–570 nm^22,31–40^. To the best of our knowledge, this study showcases impressive EQE performance, even when dealing with small emission areas. However, the efficiency droop phenomenon occurs when the current density is over 20 A/cm^2^. The significant drop in efficiency observed when operating micro-LEDs at high injected current densities was linked to the Auger recombination^41^. It is worth noting that the efficiency droop of the yellow micro-LEDs is slightly lower than the red ones. The variance in EQE maximum and efficiency droop between the yellow and red micro-LEDs can be attributed to the disparity in their indium content, with the higher indium content resulting in more severe QCSE.

### Modulation bandwidth and transmission data rate

**Figure 5:**
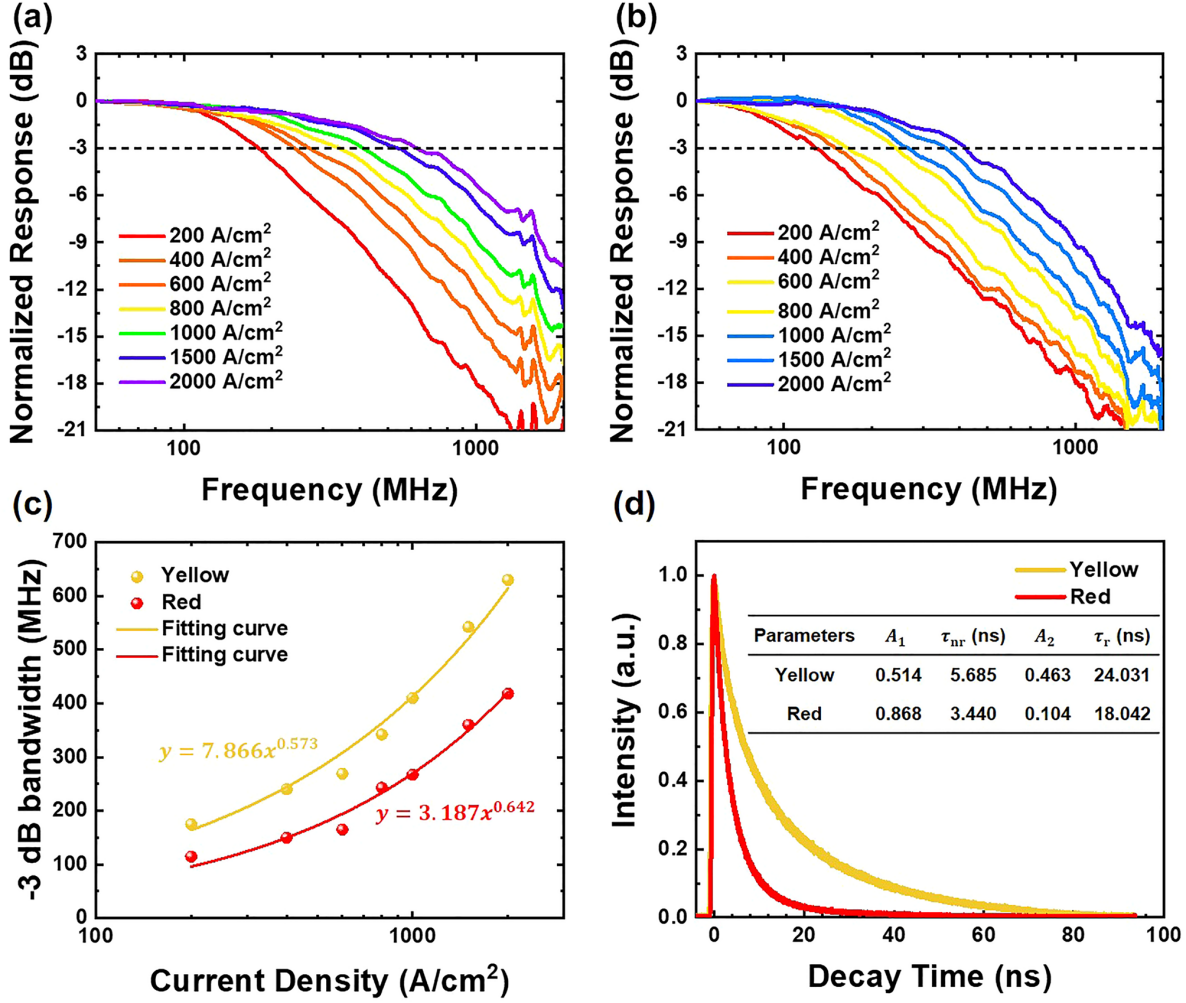
Normalized frequency response for (**a**) yellow micro-LEDs and (**b**) red micro-LEDs. (**c**) Frequency versus current density for yellow and red micro-LEDs. (**d**) TRPL curves micro-LEDs at room temperature.

In a VLC system, bandwidth stands out as a crucial metric since it directly influences the transmission rate. The frequency response of LEDs is influenced by carrier dynamics and parasitic capacitance. These characteristics are represented by the carrier lifetime and the RC time constant. However, for micro-LEDs with dimensions of 100 × 100 μm^2^ or smaller, the relatively tiny geometrical capacitance plays a vital role in preventing the RC time constant from becoming a major limiting factor in device performance^42^. Therefore, the modulation bandwidth is notably affected by the carrier recombination lifetime, rendering it a pivotal parameter. The − 3 dB bandwidth of the micro-LEDs can be expressed as:

2 $${f}_{-3\,\mathrm{ dB}}\propto \frac{1}{\tau }$$

where ${f}_{-3\,\mathrm{ dB}}$ and $\tau$ represent modulation − 3 dB bandwidth and carrier lifetime, respectively. To assess the micro-LEDs’ frequency response, we employed a vector network analyzer (VNA, HP8720ES). A bias tee connected the VNA-generated alternating current signal to a direct current, and we introduced this coupled signal into the micro-LEDs using a microscope (ACP40-GS-250), resulting in a low-amplitude optical signal. Subsequently, we utilized a plastic optical fiber to collect and transmit the optical signals from the micro-LEDs to a photodetector (Graviton, SPA-3). The photodetector then converted these optical signals into electrical signals, enabling us to analyze the frequency response with the VNA. Figure 5a,b illustrate the frequency response of the yellow and red micro-LEDs, respectively. It can be observed that as the current density increases, the bandwidth also increases. The maximum value of bandwidth is reached at a current density of 2000 A/cm^2^. Specifically, the yellow micro-LEDs achieved a maximum − 3 dB bandwidth of 630 MHz, while the red devices reached 418 MHz.

To delve deeper into the analysis, if EQE remains relatively stable with changes in current density. In doing so, we can establish a relationship, denoted as ${f}_{-3\,\mathrm{ dB}}\propto {J}^{m}$, where $m$ represents a power factor varying from 0 to 1. When the radiative recombination predominantly influences the modulation bandwidth, $m$ tends to approach a value around 0.5^23,43^. If $m$ is less than 0.5, the modulation bandwidth gradually succumbs to Shockley–Read–Hall (SRH) recombination. Conversely, if $m$ exceeds 0.5, it signifies that the Auger recombination takes over the bandwidth. We utilized these characteristics to determine the prevailing recombination mechanism. Figure 5c depicts the correlation between current density and bandwidth for yellow and red micro-LEDs, with power factors of 0.573 for the yellow and 0.642 for the red, as determined through fitting. This observed outcome, primarily influenced by Auger recombination, serves to substantiate the underlying factors contributing to the decrease in EQE performance efficiency. In addition, it implies that the red micro-LEDs likely involve a greater contribution of non-radiative recombination mechanisms due to the more pronounced QCSE, giving rise to less wavefunction overlap.

We then utilize time-resolved photoluminescence (TRPL) measurements to determine carrier lifetimes. These measurements provide valuable insights into the dynamics of carriers within semiconductor materials, shedding light on both radiative and non-radiative processes. Figure 5d shows the TRPL spectra of both epitaxial samples at room temperature. The decay curves were fitted using a bi-exponential function as follows:

3 $$I\left(t\right)={A}_{1}{\text{exp}}\left(-t/{\tau }_{{\text{nr}}}\right)+{A}_{2}{\text{exp}}\left(-t/{\tau }_{{\text{r}}}\right)$$

where $I\left(t\right)$ is the TRPL intensity at time $t$, ${\tau }_{{\text{nr}}}$ and ${\tau }_{{\text{r}}}$ represent the non-radiative recombination and radiative recombination lifetime, respectively^44^. The coefficient preceding the exponential term is linked to the quantity of carriers undergoing the recombination process. The fitting results are shown in the inset of Fig. 5d. Surprisingly, considering that the carrier lifetimes in the yellow sample are noticeably longer in comparison to those in the red sample, there is an intuition that the frequency response of yellow micro-LEDs should be lower. However, this assumption contradicts the actual outcomes of our frequency response measurements. We believe that when comprehensively analyzing the frequency response, it is essential to consider both ${A}_{1}$ and ${A}_{2}$ coefficients. Even though red micro-LEDs have a faster recombination rate, the probability of these carriers recombining into photons is significantly diminished. Therefore, this reduced likelihood of carrier-to-photon recombination has an impact on the overall performance of the frequency response, leading to a lower response when compared to their yellow micro-LED counterparts. Additionally, based on the TRPL measurement, it is evident that the majority of carriers in both samples are governed by the non-radiative recombination process, consistent with the findings from the relationship between the bandwidth and current density.

Optical communication transmission is a cutting-edge technology that utilizes optical signals to convey information, whether through optical fibers or free space. It is a high-speed, broadband data transmission method that has evolved into a crucial component of modern communication systems. An optical communication system typically comprises several key elements: a light source, a modulator, a fiber optic transmission medium, and a receiver. In this study, we employ micro-LEDs as the light source. The signals are generated by a microwave signal generator and subsequently modulated onto the micro-LEDs using a bias tee. These micro-LEDs play a pivotal role in converting the signal into changes in light intensity, frequency, or phase. The light, now carrying the information of the signals, is transmitted through the optical fibers to a photoelectric detector. Optical fibers serve as the primary transmission medium in optical communication, utilizing total internal reflection to propagate optical signals. The optoelectronic detector at the receiving end then transforms these optical signals back into electrical signals. To ascertain the integrity of the transmitted information, an oscilloscope is employed to restore the original content. The performance of the transmission is then visually depicted and observed in the form of eye diagrams.

**Figure 6:**
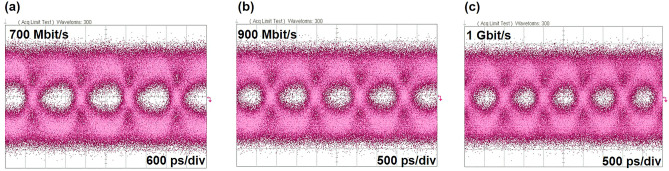
NRZ-OOK eye diagram for 30 μm × 8 yellow micro-LED arrays at (**a**) 700 Mbit/s, (**b**) 900 Mbit/s, and (**c**) 1 Gbit/s.

Since yellow micro-LEDs have achieved a higher − 3 dB bandwidth, it is worthwhile to investigate their potential transmission data rate. We initially assessed the transmission performance of these micro-LEDs using an off–on keying (OOK) system using a non-return-to-zero (NRZ) 2^7 ^− 1 pseudorandom binary sequence (PRBS7) generated by a bit pattern generator (Anritsu MP1800A). Figure 6 shows the NRZ-OOK eye diagrams of yellow micro-LEDs driven at a current density of 2000 A/cm^2^, with an applied signal transmission rate ranging from 700 Mbit/s to 1 Gbit/s. As evident from the figure, the yellow micro-LEDs exhibit a clear and open eye pattern at a data rate of 700 Mbit/s. The clarity of the eye diagram at this data rate showcases the robust performance of the micro-LEDs, indicating a significant margin for error-free data transmission. As the data rate escalates to 900 Mbit/s, the eye gradually begins to close, signifying a narrowing of the margin between signal levels, which can potentially impact the reliability of data reception. When operating at a data rate of 1 Gbit/s, though the eye diagram tends to reach its transmission limit, it’s crucial to highlight that the bit error rate (BER) stands at 2.1 × 10^–3^, which falls well within the bounds of the forward error correction (FEC) limit of 3.8 × 10^–3^.

**Figure 7:**
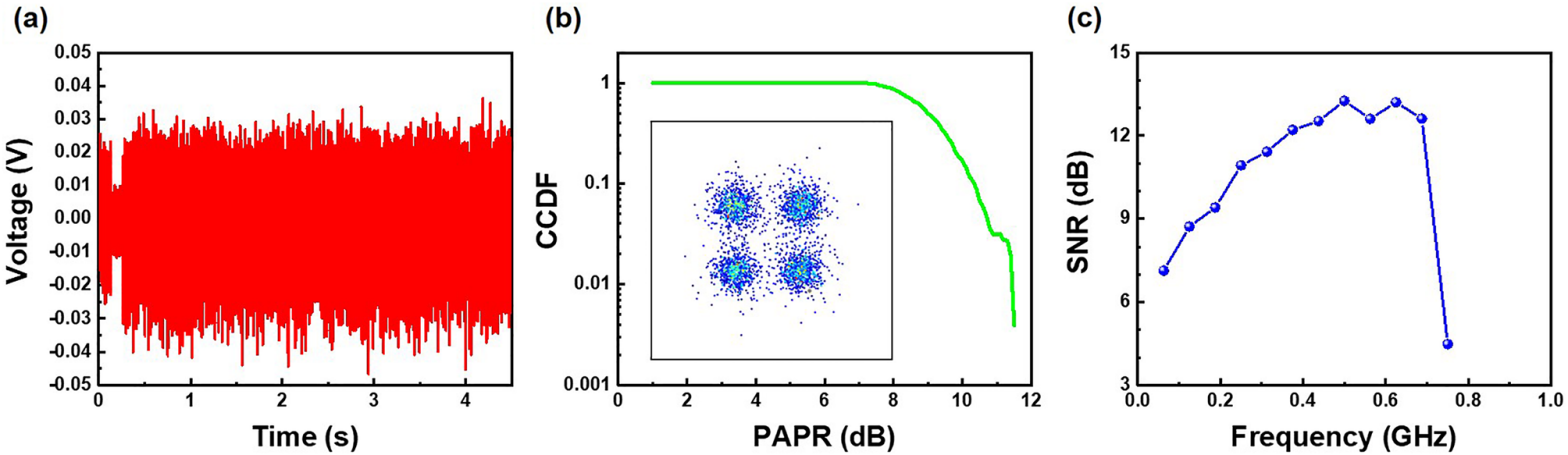
OFDM measurement. (**a**) Waveform, (**b**) CCDF of PAPR, and (**c**) SNR spectrum of the optical 4-QAM OFDM data with a modulation bandwidth of 750 MHz for yellow micro-LED arrays. The constellation plot is shown in the inset of this figure (**b**).

When subject to high current density, the yellow micro-LEDs experience the band-filling effect and piezoelectric field screening effect, causing a shift in wavelength toward the green emission band. A benchmark of VLC performance for yellow-green micro-LEDs is summarized in Table 1^20,22,45–52^. To the best of our knowledge, this work has achieved the highest − 3 dB bandwidth for yellow-green micro-LEDs to date. Regarding the transmission data rate, it’s worth noting that there is potential for achieving a higher transmission data rate by employing alternative modulation formats. Consequently, the OFDM measurements have been employed to further enhance the data rates. In the process of synthesizing the electrical 4-quadrature amplitude modulation (QAM) OFDM data stream, we configured the data bandwidth to be 750 MBaud, and the resulting received data stream is represented in Fig. 7a–c. Upon close examination of the received data waveform, it becomes evident that there is a slight disparity in amplitude between the positive and negative segments, measuring approximately + 0.3 V and − 0.4 V, respectively. This asymmetry in amplitude can be attributed to power saturation within the yellow micro-LED, primarily stemming from the drooping effect that occurs during operation with a high bias^21^. Analyzing the 4-QAM OFDM constellation plot reveals an error vector magnitude (EVM) of 33.6%, a peak-to-average power ratio (PAPR) of 10.3 dB at a complementary cumulative distribution function (CCDF) of 0.1, and the SNR spanning from 13 to 3 dB across the 750 MHz data bandwidth. Moreover, the decoded BER of 1.47 × 10^–3^ falls comfortably below the FEC threshold of 3.8 × 10^–3^. This indicates that the yellow micro-LED is capable of achieving data rates that surpass 1.5 Gbit/s (750 MBaud) when utilizing the OFDM format. These findings collectively underscore the impressive data transmission capabilities of the yellow micro-LED, particularly in the context of the OFDM format, setting the stage for high-speed data rates and further advancements in optical communication technology.

**Table 1:** Benchmark of VLC performance for yellow-green micro-LEDs.

Structure	− 3 dB bandwidth (MHz)	Modulation format	Data rate	Year	References
72 μm 16 × 16 array	103.1	NRZ-OOK	250 Mbps	2012	^45^
–	238	NRZ-OOK	650 Mbps	2016	^46^
600 × 600 μm^2^	300	QAM-DMT	2.175 Gbps	2018	^47^
600 × 600 μm^2^	–	OFDM	2.4344 Gbps	2019	^48^
600 × 600 μm^2^	600	BL-DMT	2.7885 Gbps	2019	^49^
330 × 330 μm^2^	350	–	–	2020	^50^
–	266	OFDM	1.25 Gbps	2020	^51^
300 × 300 μm^2^	350	OFDM	3.72 Gbps	2021	^52^
–	500	QAM-OFDM	0.82 Gbps	2022	^20^
30 μm 2 × 4 array	442	NRZ-OOK	800 Mbps	2022	^22^
30 μm 2 × 4 array	630	NRZ-OOK	1 Gbps	2023	This work
50 μm 2 × 3 array		ODFM	1.5 Gbps

## Conclusion

In conclusion, we have successfully fabricated both yellow and red micro-LEDs with improved performance by precisely adjusting the indium content in the quantum wells (QWs). The superior epitaxial quality is primarily attributed to the reduced impact of the quantum-confined Stark effect (QCSE), and thus the electroluminescence (EL) measurements revealed a narrower full width at half maximum (FWHM) and a minimal wavelength shift. Considering their compact emission areas, both yellow and red micro-LEDs exhibited remarkable performance with maximum external quantum efficiency (EQE) values of 11.56% and 5.47%, respectively. In terms of visible light communication (VLC) performance, the yellow micro-LEDs showcased an impressive − 3 dB bandwidth of 630 MHz, while the red micro-LEDs achieved 418 MHz at a current density of 2000 A/cm^2^. For the data transmission, the yellow micro-LED arrays achieved 1 Gbit/s for non-return-to-zero on–off keying (NRZ-OOK) format, and an even higher 1.5 Gbit/s for orthogonal frequency-division multiplexing (OFDM) format. These results highlight the immense potential of long-wavelength InGaN-based micro-LEDs for VLC applications with exciting prospects.

## Data Availability

The data presented in this study are available from the corresponding author upon reasonable request.
